# Polylysine for skin regeneration: A review of recent advances and future perspectives

**DOI:** 10.1002/btm2.10261

**Published:** 2021-11-05

**Authors:** Payam Zarrintaj, Sadegh Ghorbani, Mahmood Barani, Narendra Pal Singh Chauhan, Mohsen Khodadadi Yazdi, Mohammad Reza Saeb, Joshua D. Ramsey, Michael R. Hamblin, Masoud Mozafari, Ebrahim Mostafavi

**Affiliations:** ^1^ School of Chemical Engineering Oklahoma State University Stillwater Oklahoma USA; ^2^ Interdisciplinary Nanoscience Center (iNANO) Aarhus University Aarhus Denmark; ^3^ Medical Mycology and Bacteriology Research Center Kerman University of Medical Sciences Kerman Iran; ^4^ Department of Chemistry, Faculty of Science Bhupal Nobles' University Udaipur Rajasthan India; ^5^ School of Chemistry, College of Science University of Tehran Tehran Iran; ^6^ Department of Polymer Technology, Faculty of Chemistry Gdańsk University of Technology Gdańsk Poland; ^7^ Laser Research Centre, Faculty of Health Science University of Johannesburg South Africa; ^8^ Department of Tissue Engineering & Regenerative Medicine, Faculty of Advanced Technologies in Medicine Iran University of Medical Sciences Tehran Iran; ^9^ Stanford Cardiovascular Institute Stanford University School of Medicine Stanford California USA; ^10^ Department of Medicine Stanford University School of Medicine Stanford California USA; ^11^ Present address: Lunenfeld‐Tanenbaum Research Institute Mount Sinai Hospital, University of Toronto Toronto ON Canada.

**Keywords:** cationic polymer, polyelectrolyte, poly‐l‐lysine, regenerative medicine, tissue engineering

## Abstract

There have been several attempts to find promising biomaterials for skin regeneration, among which polylysine (a homopolypeptide) has shown benefits in the regeneration and treatment of skin disorders. This class of biomaterials has shown exceptional abilities due to their macromolecular structure. Polylysine‐based biomaterials can be used as tissue engineering scaffolds for skin regeneration, and as drug carriers or even gene delivery vectors for the treatment of skin diseases. In addition, polylysine can play a preservative role in extending the lifetime of skin tissue by minimizing the appearance of photodamaged skin. Research on polylysine is growing today, opening new scenarios that expand the potential of these biomaterials from traditional treatments to a new era of tissue regeneration. This review aims to address the basic concepts, recent trends, and prospects of polylysine‐based biomaterials for skin regeneration. Undoubtedly, this class of biomaterials needs further evaluations and explorations, and many critical questions have yet to be answered.

**Abbreviations**
Arg: arginine
Asn: asparagine
Asp: aspartic acid
ECM: extracellular matrix
FA: focal adhesion
FAK: focal adhesion kinase
Glu: glutamic acid
Gly: glycine
HA: hyaluronic acid
hADSC: human adipose‐derived stem cell
Hep: heptose
Ile: isoleucine
IM: internal membrane
Kdo: ketodeoxyoctonate
L‐b‐L: layer‐by‐layer assembly
Leu: leucine
LSGs: lineage‐specific genes
Lys: lysine
MEF: mouse embryo fibroblasts
MSCs: mesenchymal stem cells
NCAs: N‐carboxyanhydrides
OM: intact outer membrane
PAMAM: poly(amidoamine)
PEC: polyelectrolyte complexes
PECs: polyelectrolyte complexes
PEI: polyethyleneimine
PG: peptidoglycan layer
PLL: poly‐l‐lysine
PLGA: PLL‐coated poly(lactide‐co‐glycolide)
PEM: polyelectrolyte multilayers
Pro: proline
PTK2: protein tyrosine kinase 2
ROCK: Rho‐associated protein kinase
PPP: pentose phosphate pathway
PEP: phosphoenolpyruvate
OAA: oxaloacetate
Ser: serine
Thr: threonine
Trp: tryptophan
Tyr: tyrosine
Val: valine
α‐KG: α‐ketoglutarate
ε‐PL: ε‐poly‐l‐lysine

## 1 INTRODUCTION

The ultimate goal of tissue engineering is to develop materials that can mimic the natural properties of tissue, either as a substitute for tissue repair or to encourage regeneration. Tissues damage can occur due to various causes, such as accidents and disease. The materials utilized for tissue regeneration should mimic the natural behavior of tissue and possess suitable mechanical properties and architectural structure. In this regard, several sophisticated molecules and materials have been designed to meet these requirements. The design of scaffolds for tissue engineering is a tradeoff between the complexity of the molecules and the simplicity of the application, and requires profound knowledge about the relationship between material science and biology.^1^, ^2^, ^3^ Polymers have attracted significant attention in the realm of regenerative medicine, because of their unique properties and chemical versatility. These macromolecules can be synthesized as various chemical and physical structures resulting in wide range of physicochemical properties. For example, polymers can be designed to meet the requirements for mimicking soft tissue (e.g., skin) and for hard tissue (e.g., bone).^4^, ^5^ In this regard, many natural and synthetic polymers, such as chitosan,^6^, ^7^ gelatin,^8^, ^9^ and agarose^10^, ^11^ have been investigated for tissue regeneration because of their beneficial properties.

These polymers can be categorized based on their charge, as neutral, cationic, or anionic polymers. For example, most of the polysaccharides are anionic polymers due to the presence of pendant anionic functional groups (e.g., hydroxyl and carboxyl) on their main chains. On the other hand, cationic polymers are positively charged polyelectrolytes, carrying positive charges either on their backbone or on the side chains, and have attracted much interest among the polymer families. Cationic polymers have been more often used in biomedical applications compared to anionic polymers, because of their interaction with negatively charged biomolecules, peptides, proteins, and nucleic acids. Polycationic polymers can interact with negatively charged cell membranes and enhance cellular activities.^12^, ^13^, ^14^, ^15^, ^16^ Polycationic polymers such as poly‐l‐lysine (PLL), polyethyleneimine (PEI), and poly(amidoamine) (PAMAM) dendrimers have been widely utilized to carry cargos (e.g., drugs, genes, and so on) across the cell membrane.^17^, ^18^, ^19^ They can be used to form polyelectrolyte complexes (PECs) by combining with anionic species such as polyanions, nucleic acid chains, and some drugs.^20^ Besides, they can form layer‐by‐layer assemblies.^21^ Moreover, some polycationic polymers exhibit intrinsic bioactivity, with antibacterial, antioxidant, and antitumor properties.^22^, ^23^

Polylysine is a cationic polymer, which has attracted significant attention in tissue engineering because of its biodegradability and biocompatibility. However, because of its poor mechanical properties, it is not often used alone, and it should be copolymerized or blended with other polymers to improve its mechanical properties. Controlled polymerization, grafting, and modification techniques have been used to prepare PLL‐based materials with the desired architecture, properties, and multi‐functionality as biomimicking scaffolds for tissue engineering applications.^22^ Due to the unmet need for skin tissue regeneration and considering the adjustable and unique properties of the polylysine, it is necessary to review the polylysine performance in skin tissue engineering.

## 2 THE CHEMISTRY OF POLYLYSINE

**FIGURE 1:**
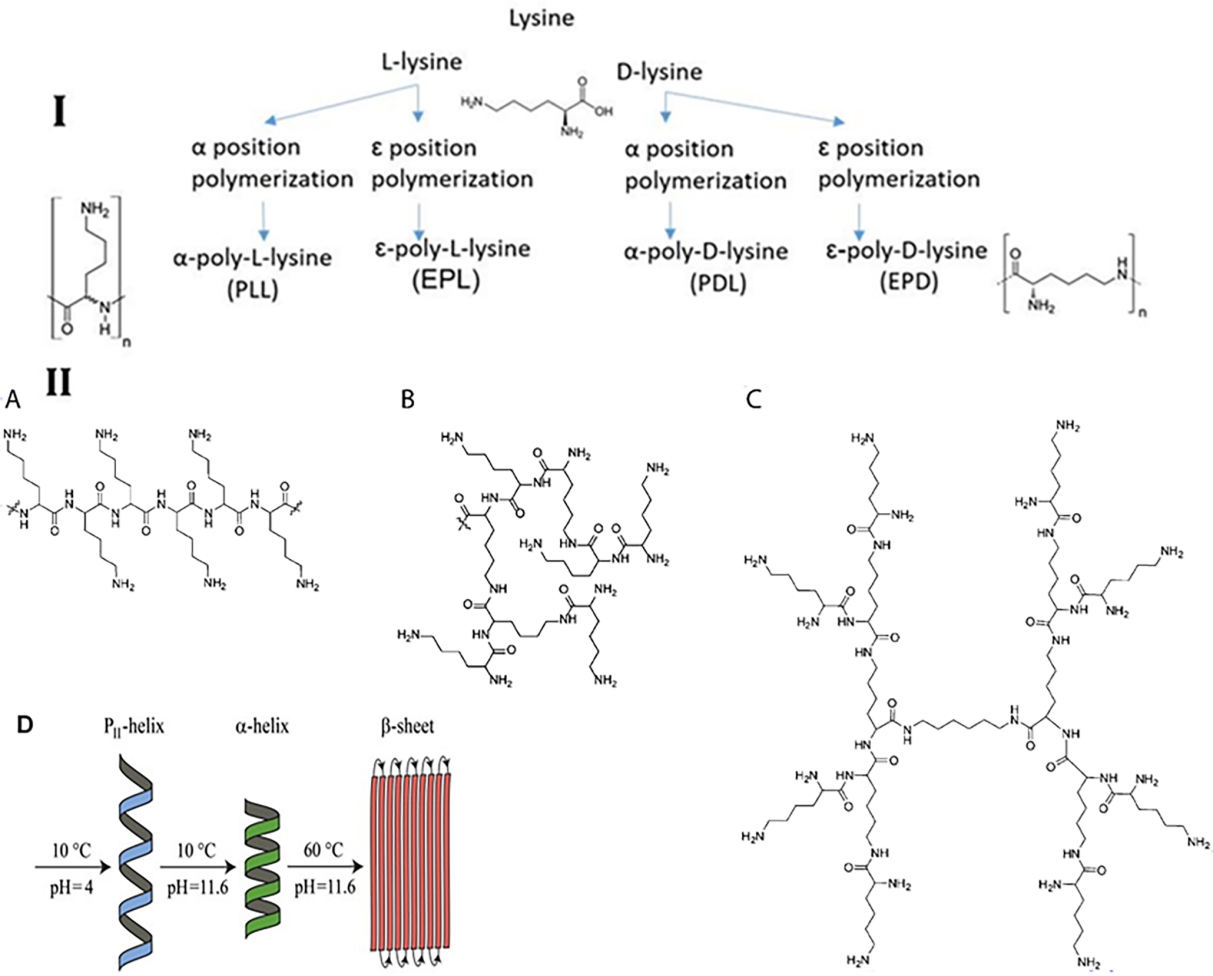
Polylysine structures. I. Lysine structure and polylysine formation. II. Polylysine structure (A) linear, (B) dendritic, (C) hyper‐branch, and (D) conformation of the polylysine at different temperatures and pH. Reproduced with permission from Reference [24]

Lysine (an essential amino acid for humans) is the primary building block of polylysine, and is available in two chiral forms: l‐lysine and d‐lysine. Polylysine can be synthesized through condensation polymerization or by fermentation, which results in α‐polylysine or ε‐polylysine, respectively. ε‐Polylysine allows the linking of ε‐amino and α‐carboxylic acid moieties and is utilized as a food preservative due to its nontoxicity and antibacterial properties. The conformation of poly‐l‐lysine (PLL) depends on pH, temperature, ionic strength, and solvent type.^24^, ^25^ PLL in aqueous solution exhibits the P_II_ conformation at low temperature and neutral pH, while by raising the pH the conformation changes to an α‐helical conformation, and by raising the temperature it changes to a β‐sheet structure. It is thought that the steric interactions between the PLL side chains (uncharged), the hydrogen bonds of the solvent or PLL, and the proton donor and acceptor groups in the solvent, stabilize the P_II_ conformation.^24^ The different forms of lysine and their different polymerization structures are depicted in Figure 1.

**FIGURE 2:**
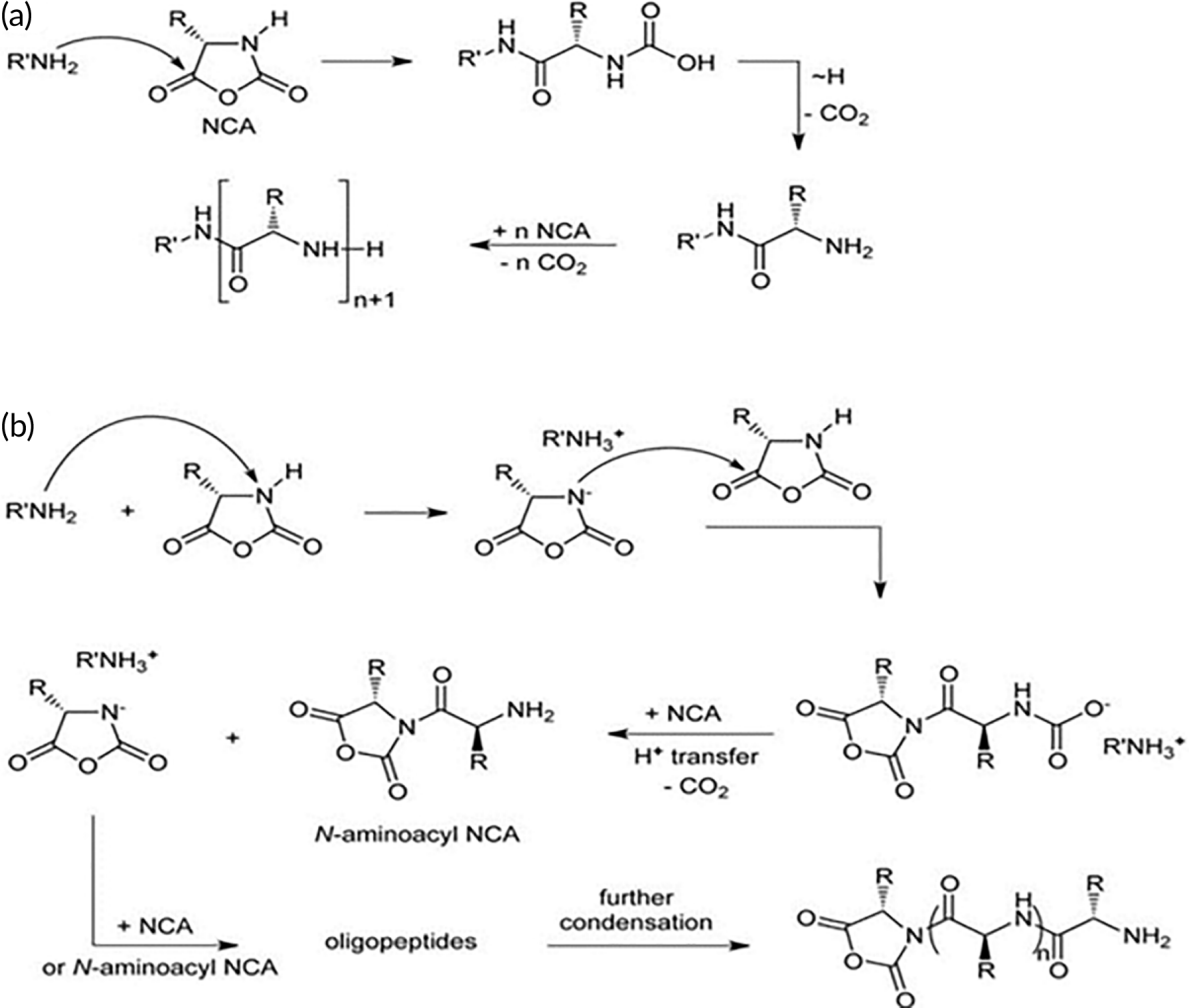
Synthesis of poly‐l‐lysine (PLL). PLL was synthesized using the conversion of the ε‐primary amine‐protected l‐lysine into the cyclic N^ε^‐(benzyloxycarbonyl)‐l‐lysine N‐carboxyanhydride monomer. NCA via ring‐opening polymerization was polymerized starting by primary amine initiator which can operate in two ways by (A) attacking the C5 of NCA as a nucleophile (amine mechanism) and (B) deprotonate the N_3_ of NCA as a base (active monomer mechanism)

Amino acid N‐carboxyanhydrides (NCAs), which are also known as leuchs anhydrides are well‐known reactive amino acid derivatives.^26^ Ring‐opening polymerization of NCAs is a useful method for polypeptide synthesis because of its good yield.^27^, ^28^ PLL has been synthesized using the conversion of and ε‐primary amine‐protected l‐lysine derivative into the cyclic N^ε^‐(benzyloxycarbonyl)‐l‐lysine N‐carboxyanhydride monomer. NCA was polymerized via ring‐opening polymerization starting with a primary amine initiator which can operate in two ways: (1) attacking the C5 of NCA as a nucleophile (amine mechanism); and (2) deprotonating the N_3_ of NCA as a base (active monomer mechanism). Moreover, living polymerization using metal complexes has been used for NCA polymerization (Figure 2).^22^, ^29^

## 3 INTERACTIONS BETWEEN CELLS AND POLYLYSINE SUBSTRATES

### 3.1 Simple polylysine substrates

The interactions between cells and their neighboring environment play a key role in governing cellular activity, such as during the processes of morphogenesis, embryogenesis, homeostasis, thrombosis, inflammation, and wound healing.^30^, ^31^ The extracellular matrix (ECM) binding‐site affects the shape of the cell and its motility. The first step in cell‐substrate interaction is the binding of integrin receptors to ECM molecules, followed by the aggregation of receptors at the contact point.^32^, ^33^, ^34^ This is called the focal adhesion complex. Intracellular signaling cascades are triggered, resulting in the binding of the actin cytoskeleton to the ECM mediated by integrins.^35^, ^36^ Surface treatment of surfaces using polycationic polymers, such as polylysine, has been used to improve cell‐substrate interactions and facilitate cell adhesion.^37^

The use of polyamine acids enhances cellular attachment to the substrate. Cellular functions are affected by this attachment and by ECM components. Some cells are able to produce ECM components, while other cells require an adjuvant to attach to the surface (especially in serum‐free media). Polylysine (because of its positively charged nature) can easily interact with the negatively charged surface on many types of cells. Therefore, cells can rapidly attach to polylysine substrates. Interestingly, some cells (e.g., sea urchin eggs) that normally have difficulty to attach to solid surfaces, can adhere to the polylysine surface, as well as mammalian cells.^37^ Polylysine chains with higher molecular weights exhibit a higher tendency to bind to cell membranes. Moreover, flexible polycation chains exhibit higher cell‐binding compared to rigid chains.^38^ It has been observed that culturing mesenchymal stem cells (MSCs) on PLL‐coated substrates could preserve stemness and prevent senescence of these cells.^39^ PLL‐coated plates enhanced the growth of MSCs, and up‐regulated some genes that contribute to cell‐adhesion, cell differentiation, proliferation, and signaling.^39^ Cell‐proliferation and osteogenic differentiation were improved by porous PLL‐coated poly(lactide‐co‐glycolide) (PLGA)/hydroxyapatite (HA) scaffolds.^40^ Quirk et al. used coating with polylysine to attach the cells to the PLA surface.^41^ The optimum concentration of polylysine for cellular attachment was reported to be approximately 0.05–0.5 μg/cm^2^ while higher concentrations may result in toxicity.^42^ Sakai et al. used polylysine to improve the cellular attachment of BHK cells to a fibronectin surface.^43^

### 3.2 Patterned polylysine substrates

Besides physical and chemical cues which affect the cell behavior, other geometrical cues or anisotropic physical features (e.g., parallel microgrooves and collagen fibers) also affect the cell orientation on both two‐ (2D and three‐dimensional (3D) substrates, which is known as the contact guidance phenomenon.^44^, ^45^ Contact guidance affects the shape, adhesion, and migration of cells. Indeed, cell migration, which is a key factor in many biological processes, is affected by both ECM guidance cues (e.g., a chemical species gradient or stiffness) and by contact guidance. Contact guidance results in cancer cell movement across the neighboring ECM and then to metastasis, by a process known as intravasation.^46^, ^47^ Furthermore, this phenomenon plays a key role in the wound healing process, where platelets and fibroblasts can induce a local contraction in the wound site, resulting in ECM fibers becoming radially oriented. This local fiber orientation guides the cell migration.^48^

**FIGURE 3:**
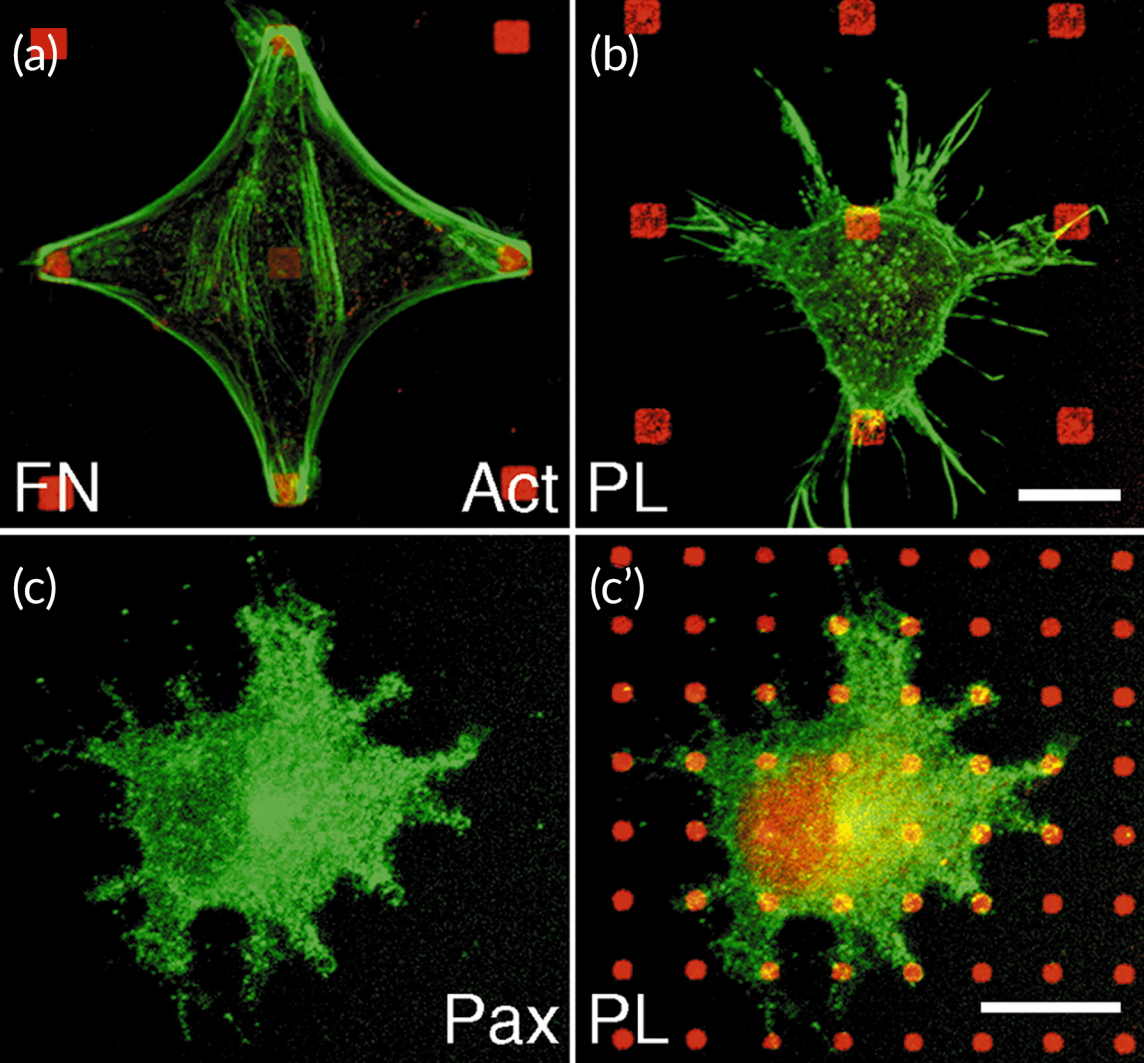
Cell spreading on polylysine. B16 cells were cultured on patterned substrata of fibronectin (red; A) or polylysine (red; B,C) and labeled for actin (green; A,B) and paxillin (green; C). Cells can adhere to polylysine dots, however, the actin cytoskeleton is not reorganized (B) and paxillin does not accumulate over the dots (C). Scale bars: 10 μm. Reprinted with permission from Reference [49]

Appropriate signaling between cells, which is mediated via ECM proteins, significantly affects the redistribution of molecules that allow cell adhesion in dot or focal patterns. Cells are able to attach to the patterned polylysine substrate, while actin stress fibers are not created between neighboring dots. Irregular morphology was observed for cultured cells in which filopodia‐like extensions were abundant; besides no clustering was observed for focal adhesion molecules.^49^ According to Lenhert et al., the cell spreading process includes three steps: (1) adhesion of the cells to the substrate, which depends on integrins; (2) integrin clustering followed by the creation of focal adhesion complexes. The focal adhesion complexes are assembled, the cells commence spreading, and Rac1 and Cdc42 are activated, resulting in the creation of lamellipodia and filopodia. The third step includes activation of RhoA, stress fiber formation, and creation of intracellular tension (Figure 3).^49^

Patterned co‐cultures can be created using three different techniques. Photolithography is a robust method to create patterned surfaces composed of different materials, that allow selective adhesion of various cells onto the predetermined area. For example, when co‐culturing hepatocytes and fibroblasts on micropatterned substrates, hepatocytes selectively adhered to the collagen islands which optimized the interaction between the different cells.^50^, ^51^ The second approach involves the delivery of cells to the specific areas on the substrate. For instance, patterning of various types of cells onto specific regions was achieved using a microfluidic device.^52^, ^53^ Furthermore, cell attachment to specific regions of the substrate can be excluded using elastomeric membranes. After removing the membrane, the remaining surface can be used to attach another different cell type.^54^ However, these strategies suffer from some limitations. The construction of multicellular systems may be limited using photolithography, as each step requires the selective attachment of a single cell type. Moreover, geometrical constraints induced by laminar flow and separation between two different cell types because of channel interspacing are limitations associated with the microfluidic method.^55^

**FIGURE 4:**
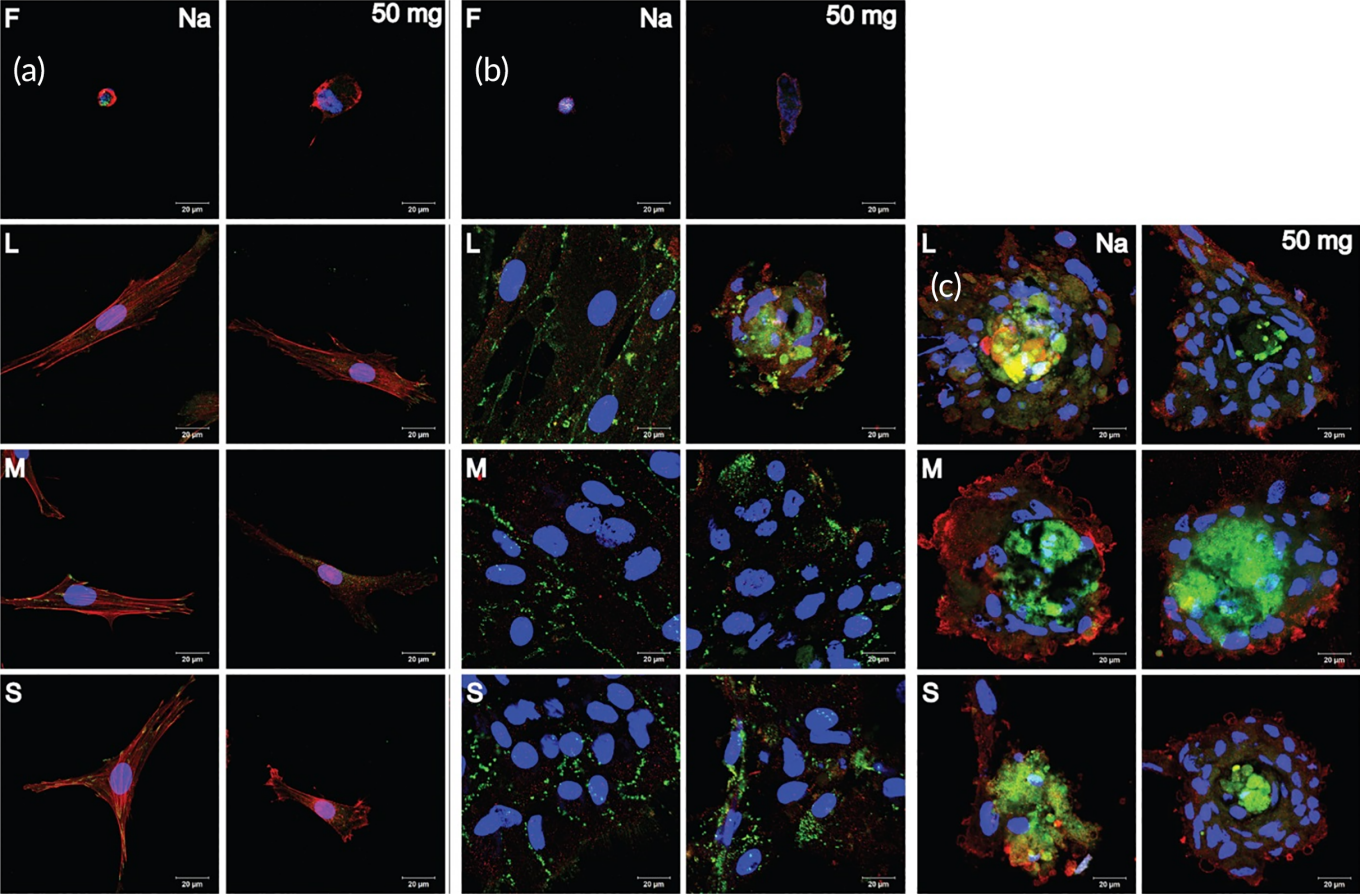
Polylysine and stem cell differentiation. (A) Confocal laser scanning microscopy images of adherent hADSC on flat (F) PEM. The platforms were cross‐linked using EDC (50, 10, and 2 mg mL^−1^). Afterward, the cells were cultured and incubated overnight (actin cytoskeleton (red), nucleus (blue), and vinculin in focal adhesions (green)). S, M, and L refer to small, medium, and large, respectively. PLL/HA and 50 mg mL^−1^ EDC cross‐linked nanostructured platforms were viewed. Nanostructured platforms were treated with PLL/HA and cross‐linked with 50 mg mL^−1^ EDC. (B) hADSC chondrogenic differentiation. (C) hADSC osteogenic differentiation. Reproduced with permission from Reference [21]

Stimuli‐responsive surfaces can be used as a general strategy to overcome these limitations; cell repelling surfaces can be switched to cell adhesive surfaces via the application of a specific stimulus.^56^, ^57^ For example, the degree of hydrophilicity of electroactive or thermosensitive substrates can be tuned to enhance or diminish cell adhesion. However, high‐cost materials and sophisticated synthetic procedures are required to prepare such switchable surfaces, and this may limit their application. Accordingly, there is an urgent need to develop improved surface engineering strategies to fabricate patterned cell co‐cultures. Layer‐by‐layer assembly (L‐b‐L) of polyelectrolytes can be potentially used to fabricate such switchable surfaces, especially thin films.^58^ Khademhosseini et al. co‐cultured either hepatocytes or embryonic stem cells with fibroblasts on a micropatterned hyaluronic acid (HA)/polylysine surface. This method was also used for patterned and controlled cell co‐culture.^59^ HA is a biocompatible and biodegradable polysaccharide that has been widely studied for targeted drug delivery and for tissue engineering applications. Many polysaccharides, including HA, naturally repel cells and many other proteins. HA is an anionic polysaccharide that can form PEC or L‐b‐L assemblies by interacting with polycations such as PLL. Niepel et al. seeded human adipose‐derived stem cells (hADSCs) on a cross‐linked polyelectrolyte based on HA plus polylysine. It was found that a higher cross‐linking density of the substrate resulted in higher cell spreading and better osteogenic differentiation, while a substrate with a lower cross‐linking density better supported adipogenic differentiation.^60^ Moreover, the hADSC fate was evaluated on the micropatterned polyelectrolyte of polylysine plus HA. When cell spreading was studied on a polyelectrolyte multilayer (PEM) with cross‐linking on a patterned substrate, there was enhanced rounding‐up of hADSCs. The patterned substrate affects both cell adhesion and differentiation, which are both important for designing novel tissue engineering scaffolds. For example, native multilayered microstructured large substrates improved chondrogenesis, while highly cross‐linked multilayered nanostructured small substrates enhanced osteogenesis (Figure 4).^21^ Richert et al. fabricated a multi‐layered polyelectrolyte film based on polylysine/poly(l‐glutamic) acid and evaluated cellular attachment. It was observed that the pH used in the film fabrication process (i.e., film deposition) affected the cell adhesion, such that cells attached more firmly to films formed at pH 10.4, while they were repelled from films formed under acidic condition, that is, pH 4.4. These observations were related to the water swelling properties of these films which were high and low for films formed under basic and acidic conditions, respectively. Accordingly, the cell adhesion performance of PGA/PLL films could be adjusted depending on the pH of the film deposition process. This study confirmed the key role of multilayered films on tuning cell attachment/detachment properties.^61^

**FIGURE 5:**
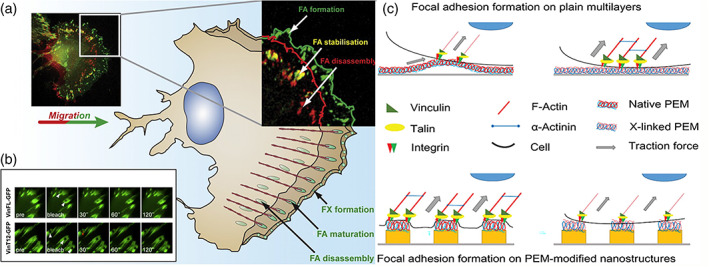
Polylysine and focal adhesions. (A) A schematic representation of the cell expressing β3‐integrin, B16 migrating melanoma, GFP, displaying, and disassembling sites of FA generation. Green mark was used to indicate fresh generated FA; structures that remained fixed within the 5‐min time intervals are labeled in yellow. (B) Fluorescence restoration of GFP vincular fusion complexes in mouse embryo fibroblasts (MEF). Reproduced with permission from Reference [62]. (C) Schematic description of the impact on the development of focal adhesion and actin polymerization of surface parameters. Reproduced with permission from Reference [21]

Focal adhesion (FA) complexes are the macromolecular assemblies responsible for the transduction of mechanical load via signaling molecules from the ECM to cell environment, that is, they serve as strain sensors or biosensors for various cells. These multiple protein structures contain integrins. Integrins are transmembrane heterodimeric proteins that connect the cells to ECM proteins (e.g., fibronectin, vitronectin, collagen, and laminin) via short amino acid sequences such as the arginylglycylaspartic acid motif. Meanwhile, they also connect the actin cytoskeleton to several proteins such as vinculin and filamin. Changes in the composition and morphology of focal adhesion complexes play a key role in cell migration. It has been proposed that the spreading of MSCs affects the cell differentiation process. The expression of lineage‐specific genes (LSGs) is influenced by phosphorylation of various gene products, such as protein tyrosine kinase 2 (PTK2) or focal adhesion kinase (FAK), and the Rho family of GTPases within the cytosol (Figure 5). MSC differentiation results in the formation of adipocytes when cell spreading is low, or osteocytes when cell spreading is high. Aspherical morphology and adipocyte differentiation have been observed in cells that were unable to undergo spreading. Furthermore, the cell morphology affects signal transduction and the shape influences signal transduction processes involving the Rho‐associated protein kinase (ROCK) pathway, resulting in adipocyte differentiation.^63^ hADSC cultivated on a highly cross‐linked platform, were stained with alizarin red showing deposition of a mineralized matrix. There was only minimal staining in cells on a lower cross‐linked or native multilayer platform in conformity with the findings of McBeath et al.^64^ This group studied stem cell distribution, RhoA activation, and cell differentiation. Cells growing on a glass substrate proliferated more than cells on a soft substrate. Consequently, the finding that during a long incubation period of 21 days, stem cells plated on a charged, rigid, glass substrate became differentiated, even with no chemical induction signals.^60^

Better knowledge about the cell‐substrate interactions allows us to understand aspects of cellular activity, such as adhesion and migration and helps us to design a biomimetic surface for the best in‐vivo function and to achieve proper tissue regeneration.

## 4 POLYLYSINE ANTIBACTERIAL ACTIVITY

### 4.1 Gram‐negative bacteria such as *Escherichia coli*

ε‐Poly‐l‐lysine (ε‐PL) exhibits the most pronounced antibacterial activity within the polylysine family, and inhibits the proliferation of most microorganisms (Table **1**). Generally, three mechanistic models including, barrel‐stave, toroidal, and the carpet mechanism have been proposed to explain the antibacterial activity of polycationic polymers like PLL. The barrel‐stave mechanism involves a rapid interaction with the hydrophobic core of the membrane lipid bilayer, while the other mechanisms rely on an interaction with the membrane surface and the headgroups. The residues with a hydrophobic nature not only can replace the headgroups of phospholipid units through a mechanism based on toroidal pore modeling, but can also induce pore lumen formation by altering the positive membrane curvature. The carpet‐like mechanism is based on a theory in which membranes are permeabilized in a nonspecific manner, involving only a contact between the positively charged peptide and the negatively charged head‐groups of the phospholipid.^71^, ^72^ The three models discussed above may not be sufficient to explain all the possible interactions within the membrane, so other models should be taken into consideration.^72^ Scientists have also tried to explain the antimicrobial activity by other models, such as the grip‐and‐dip, as well as the disordered toroidal pore model.^73^, ^74^ Moreover, many believe that some interactions could be mediated by a process analogous to electroporation, called the sinking raft model.^75^ It should be noted that in addition to the peptide structure and headgroup constitution of the membrane, the composition of existing acyl chains could also affect the peptide interaction with the membranes.^76^

**TABLE 1:** MICs of ε‐PL against parent strains and mutants of *Escherichia coli* and *Listeria innocua* detected by optical density measurements of growth at 620 nm

Strain	Phenotype	MIC (mg/L)	Ref.
D21	Parental wild‐type that express core portion of LPS	87.5	65
*E. coli*, JM109	Express full core portion of the LPS, and has an extended O‐antigen polysaccharide chain. O‐rough:H48	87.5	66
D21f2	Truncated LPS after the Kdo carboxyl group.	112.5	67
JW4277‐1	Lack type I fimbriae	87.5	68
D21f2	Truncated LPS after the Kdo carboxyl group.	112.5	69
JW5917‐1	Removed negative regulatory gene of CA synthesis. Uncontrolled	175	68, 70
*L. innocua*	Wild type	750	
*E. coli* K‐12 (LZB035)	Wild type	74	

Abbreviations: LPS, lipopolysaccharide; MIC, minimum inhibitory concentration.

**FIGURE 6:**
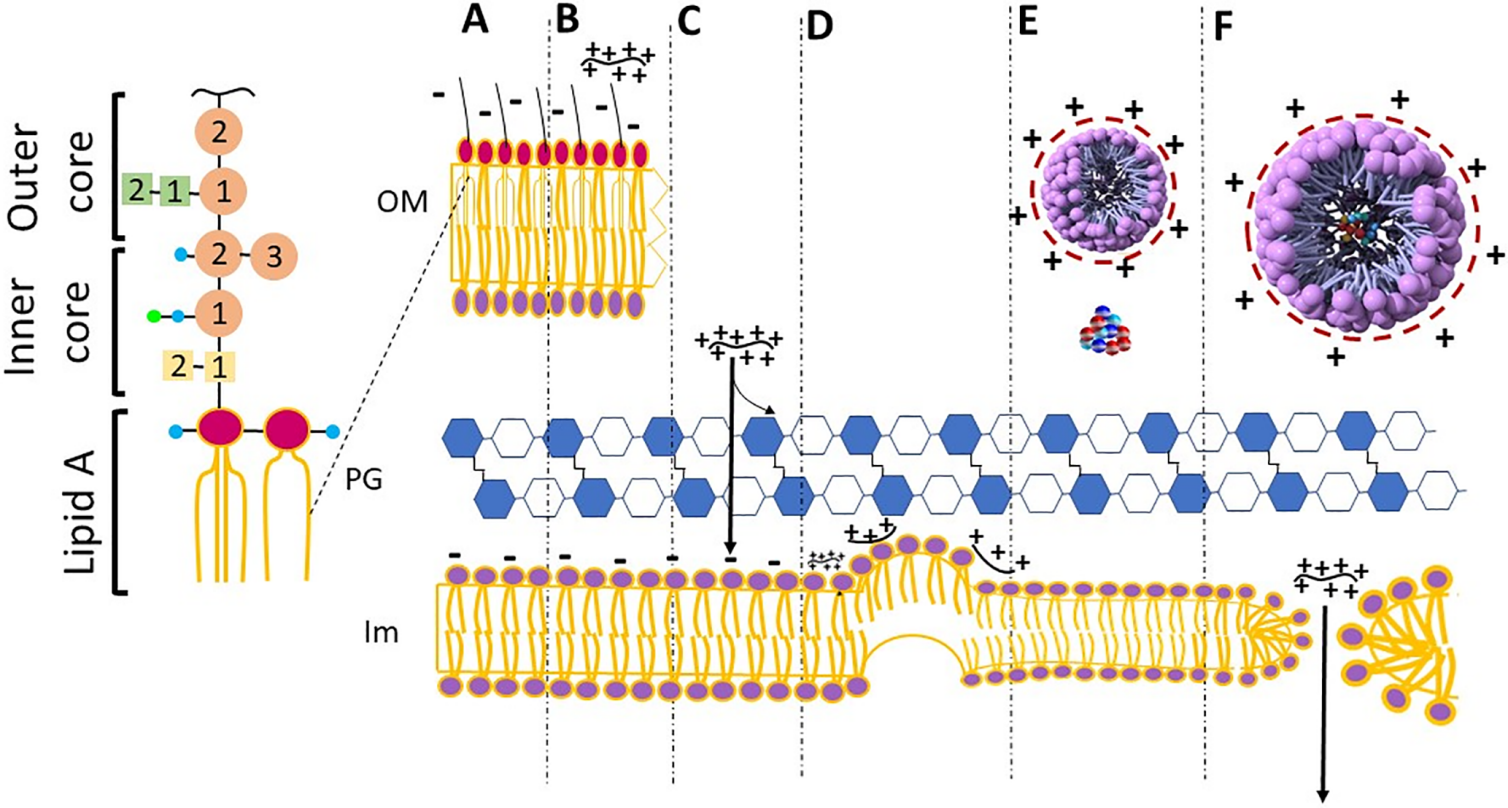
Description of the mechanisms of action suggested for ε‐PL on *E. coli* cells. (A) Intact outer membrane (OM), peptidoglycan layer (PG), and internal membrane (IM) unprotected cell wall. Lipopolysaccharide is the building blocks of the external leaflet of the external membrane. (B) The electronic interactions lead the collations of the red molecule (ε‐PL) with the LPS, which removes detergent‐like the LPS layer. (C) ε‐PL gets entry to a periplasmatic region, and there is still uncertainty about the possibility of ε‐PL interaction. (D) As ε‐PL exceeds a certain limiting value in the inner membrane, this confirms the negative curvature of one of the membrane leaflets (E) the phospholipids removed can shape hexagonal inverse micelles as well as the normal one, as indicated. (F) The phospholipids extracted from the inner membrane shape vesicles that creates holes behind where the surplus ε‐PL is released into the cytoplasm and can further damage the cell^69^

Hyldgaard et al. clarified the mechanism of the antibacterial properties of ε‐polylysine against *E. coli* as Gram‐negative and *Listeria innocua* as Gram‐positive model bacteria. It was found that some components of the *E. coli* lipopolysaccharide layer, such as the divalent cations, along with heptose (Types I and II)‐phosphate could play a role in the binding process with polylysine. Polylysine destroyed the *E. coli* lipopolysaccharide layer and altered its morphology, while *L. innocua* showed only minimal morphological changes. The membranes of both the bacteria were attacked by polylysine, as shown by cytoplasmic membrane permeabilization. The carpet‐like model could also explain how polylysine could disrupt the membrane stability. This involves the polylysine binding to negatively charged phospholipids and releasing existing divalent cations, and consequently changing the membrane curvature into negative folding, thereby producing structures similar to vesicles or micelles (Figure 6).^69^

The concentration of divalent cations in the ambient medium will competitively inhibit the binding of ε‐PL to the bacterial membranes. Moreover, an increase in the pH will decrease the positive charge on the ε‐PL, and subsequently, reduce the electrostatic interaction with the cells, and lessen the bactericidal activity. ε‐PL shows selective antimicrobial activity against a wide range of microorganisms, without any pronounced cytotoxicity towards eukaryotic cells, because the phospholipid head groups are different in eukaryotic and prokaryotic membranes.

**FIGURE 7:**
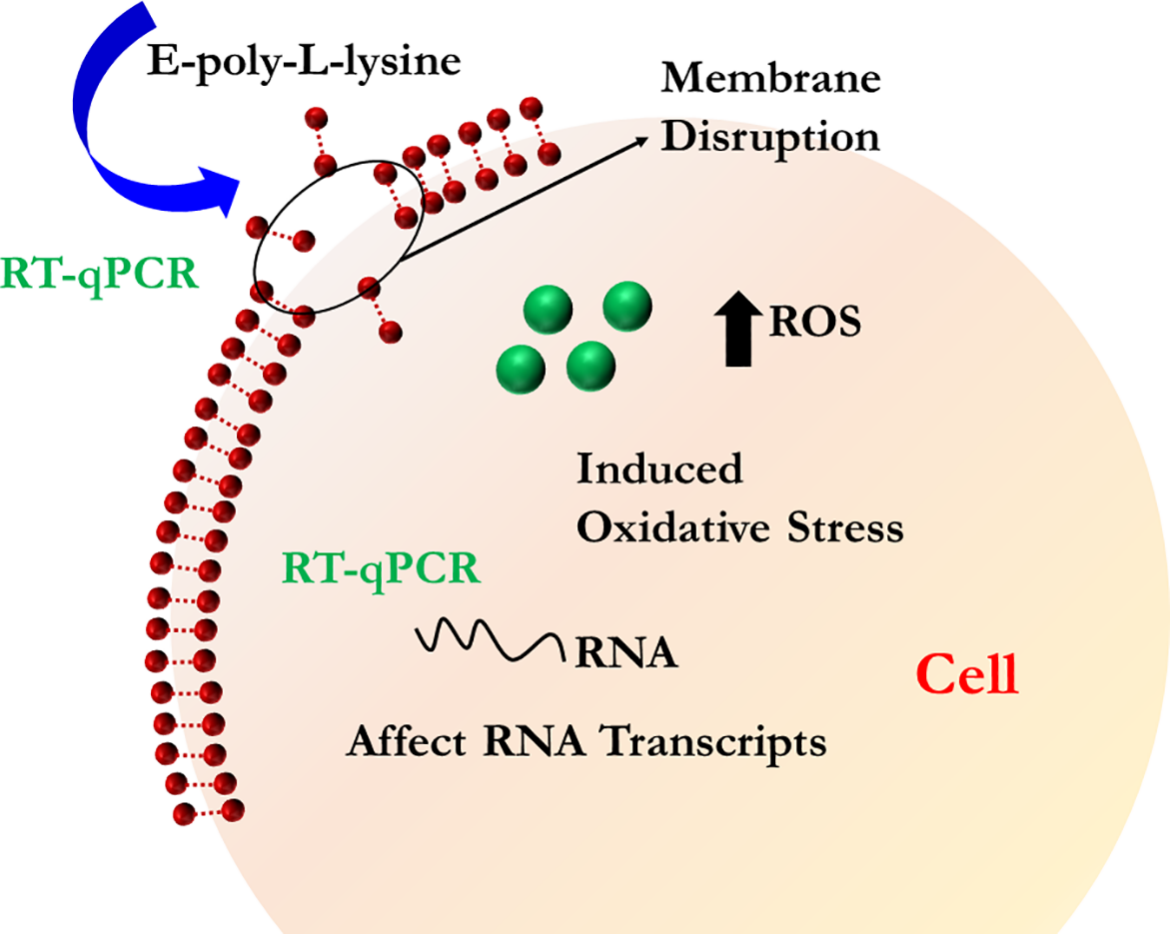
Bactericidal mechanism for ε‐PL against *Escherichia coli* O157: H7. ε‐PL bound to the membrane through the electrical forces resulting in both the destruction of the aimed membrane and formation changes and membrane fractures. The cytoplasm was entered through membrane pores by ε‐PL and two parallel reactions occurred afterward, that is, an increase in reactive oxygen species (ROS) levels and the DNA genome interactions, resulting in cell death. Reproduced with permission Reference [78]

In addition to membrane disruption, the antibacterial activity of ε‐PL could also involve other mechanisms. The formation of reactive oxygen species (ROS) and oxidative stress has been described as a result of cationic antibacterial compounds acting on cells. The findings of Del Carlo and Loeser in 2006 showed that ROS were produced by poly‐l‐lysine, and could cause substantial cell death in chondrocytes and bacteria that could be prevented by antioxidants.^77^ Various genes associated with chemotaxis and oxidative/redox stress were shown to be induced by antibacterial compounds. The genes that were activated were concerned with the biosynthesis of adenosine triphosphate and the SOS response, and were associated with antibacterial activity.^71^, ^72^ RT‐qPCR results showed that the SOS response genes *recA* and *lexA*, were triggered by up‐regulation of the bacterial‐oxidative stress response as shown by the ROS‐related genes *oxyR* and *sodA*, and a decrease in the transcription of virulence genes *eaeA* and *espA*. The possible mechanisms of membrane disruption produced by ε‐PL are shown in Figure 6. The possible mechanisms of ROS and SOS responses produced by ε‐PL are shown in Figure 7.^78^

### 4.2 Gram‐positive bacteria such as *Staphylococcus aureus*

**FIGURE 8:**
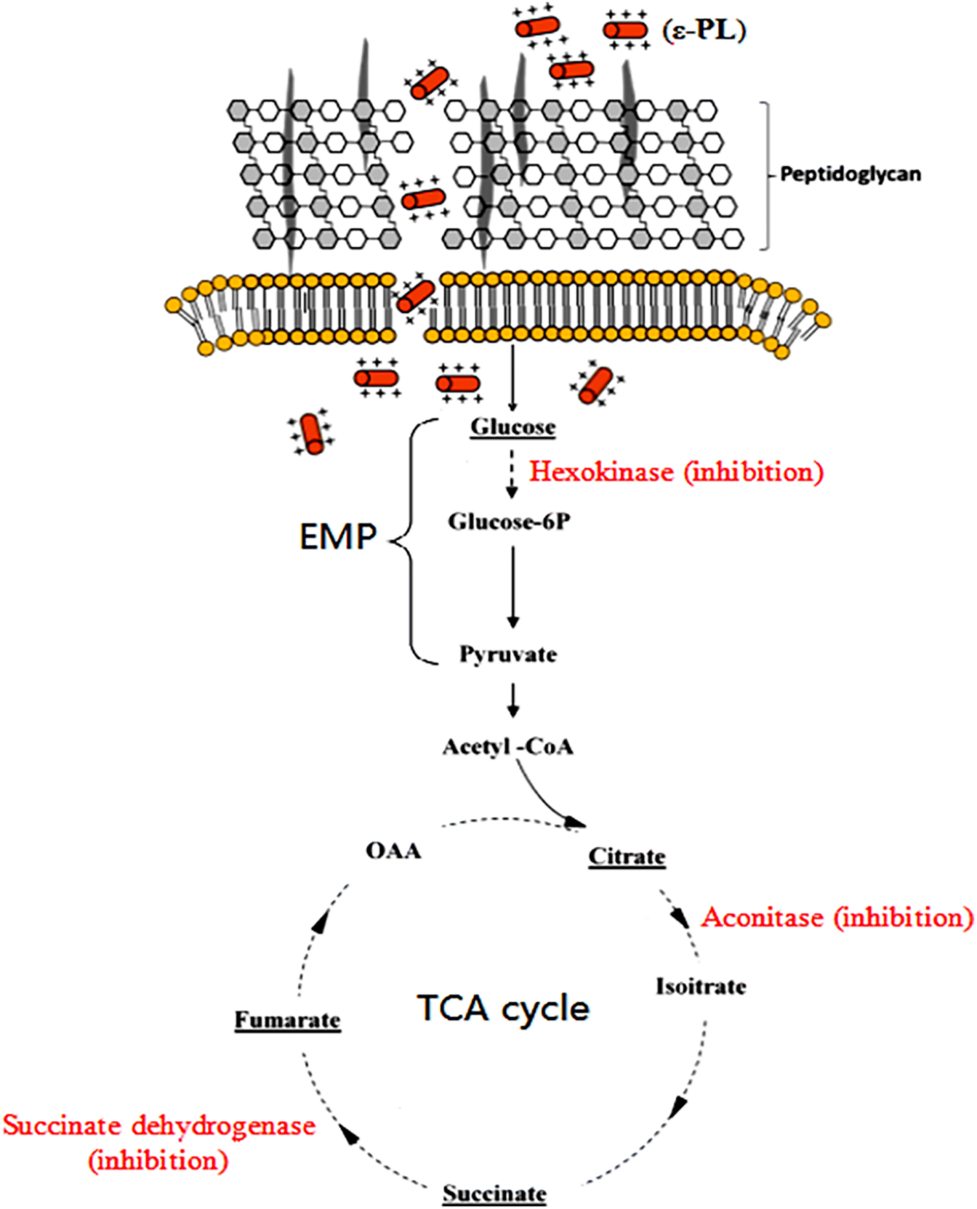
Proposed antibacterial mechanism of ε‐PL against *Staphylococcus aureus*. First, cationic ε‐PL attaches to negatively charged teichoic acid embedded in the peptidoglycan layers of the *S. aureus* cell wall. The peptidoglycan structure in the cell wall is destroyed by ε‐PL, leading to cell wall fragility. Next, the cell membrane is disturbed by ε‐PL, and this disturbance further induces the changes in hydrophobic region and bilayer curvature of cell membrane, causing increase of cell membrane permeability. Finally, ε‐PL enters into the cells and interrupts the primary metabolism of *S. aureus*; thus, killing the cells. Reproduced with kind permission from Reference [79]

The potential mechanism for the antimicrobial activity of ε‐PL against Gram‐positive bacteria such as *S. aureus*, is presented in Figure 8. In the first stage, the positively charged ε‐PL binds to negatively charged teichoic acid which is a component of the peptidoglycan layers within the cell wall of *S. aureus*. Next, ε‐PL can disrupt the cell wall peptidoglycan, causing fragility of the cell wall. The cell membrane is then disrupted by the ε‐PL, which further contributes to the disruption of the hydrophobic region and cell membrane curvature in both layers, resulting in increased permeability of the cell membrane. Lastly, ε‐PL gets inside the cells where it disrupts the main bacterial metabolism, thus, killing the cells. These mechanisms are shown in Figure 8.^79^ Because polylysine destroys bacteria via membrane disruption (barrel stave and electroporation) and perturbation (charged lipid clustering) without affecting the cytoplasm, it is thought to be unlikely to produce any antimicrobial resistance even after repeated application.^80^

### 4.3 Mycelial fungal cells such as *Penicillium digitatum*

Other have reports revealed that eukaryotic fungal cells (such as *P. digitatum*) could be inhibited by ε‐PL.^81^ ε‐PL affected mycelial production, and the spore germination rate and germ tube duration were significantly inhibited. Moreover, ε‐PL could also affect the cell wall morphology in eukaryotic fungal cells, and disrupt their cell membrane.^82^ These findings, therefore, showed that mycelial inhibition, spore development, and damage to the cell wall and membrane were all caused by ε‐PL and were linked to the death of eukaryotic fungal cells.

### 4.4 Yeast cells such as *Saccharomyces cervisiae*

**FIGURE 9:**
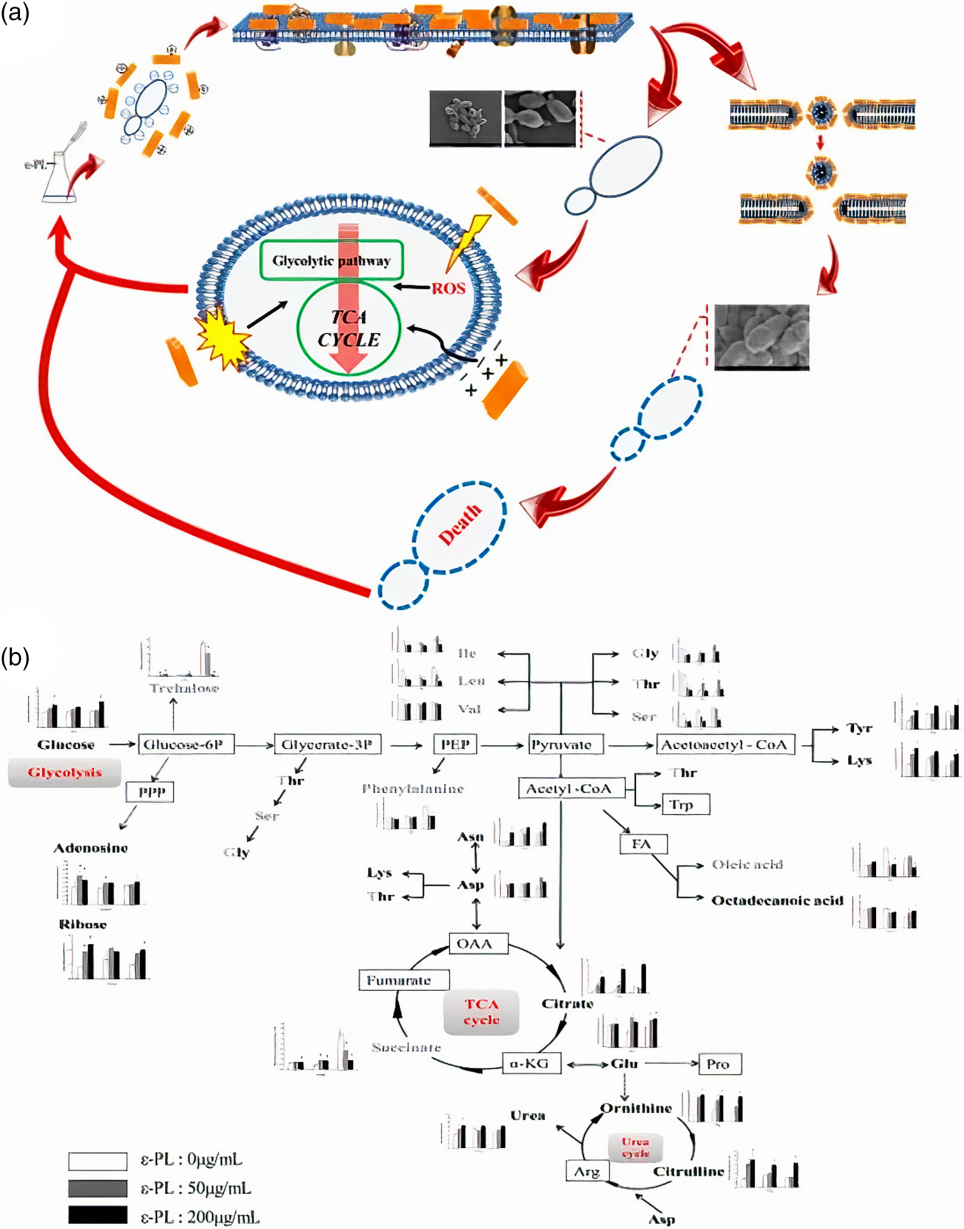
Antibacterial effects of ε‐PL versus *Saccharomyces cerevisiae*. (A) Mechanisms of action. (B) Variations in metabolite concentration at the sampling periods of 4, 8 and 12 h on the metabolic network. Reproduced with permission from Reference [83]

It was observed that the fungicidal and fungistatic activities of ε‐PL were different against yeast species, as shown for *S. cerevisiae* cells. As shown in Figure 9, the potential mechanism has been suggested for the antifungal activity of ε‐PL, which binds to components of the cell membrane by electrostatic forces. If the concentration ε‐PL exceeds a threshold value, ε‐PL will rapidly become embedded in the *S. cerevisiae* cell membrane, following the mechanism of the carpet model, leading to the development of micelles, the folding of the phospholipid bilayer, and breaking open the cell membrane and eventually leading to cell death. However, a concentration of ε‐PL lower than the threshold level could still affect the structure, fluidity, and permeability of the cell membrane. The interference with central intracellular metabolism involving the carbon growth source could also contribute to the disruption of membrane function. Simultaneously, ε‐PL will also activate the intracellular generation of ROS and induce cell apoptosis. These cumulative steps caused by ε‐PL will contribute, in most cases, to inhibition of the growth of *S. cerevisiae*.^83^

## 5 POLYLYSINE‐BASED SCAFFOLDS IN SKIN TISSUE ENGINEERING

**FIGURE 10:**
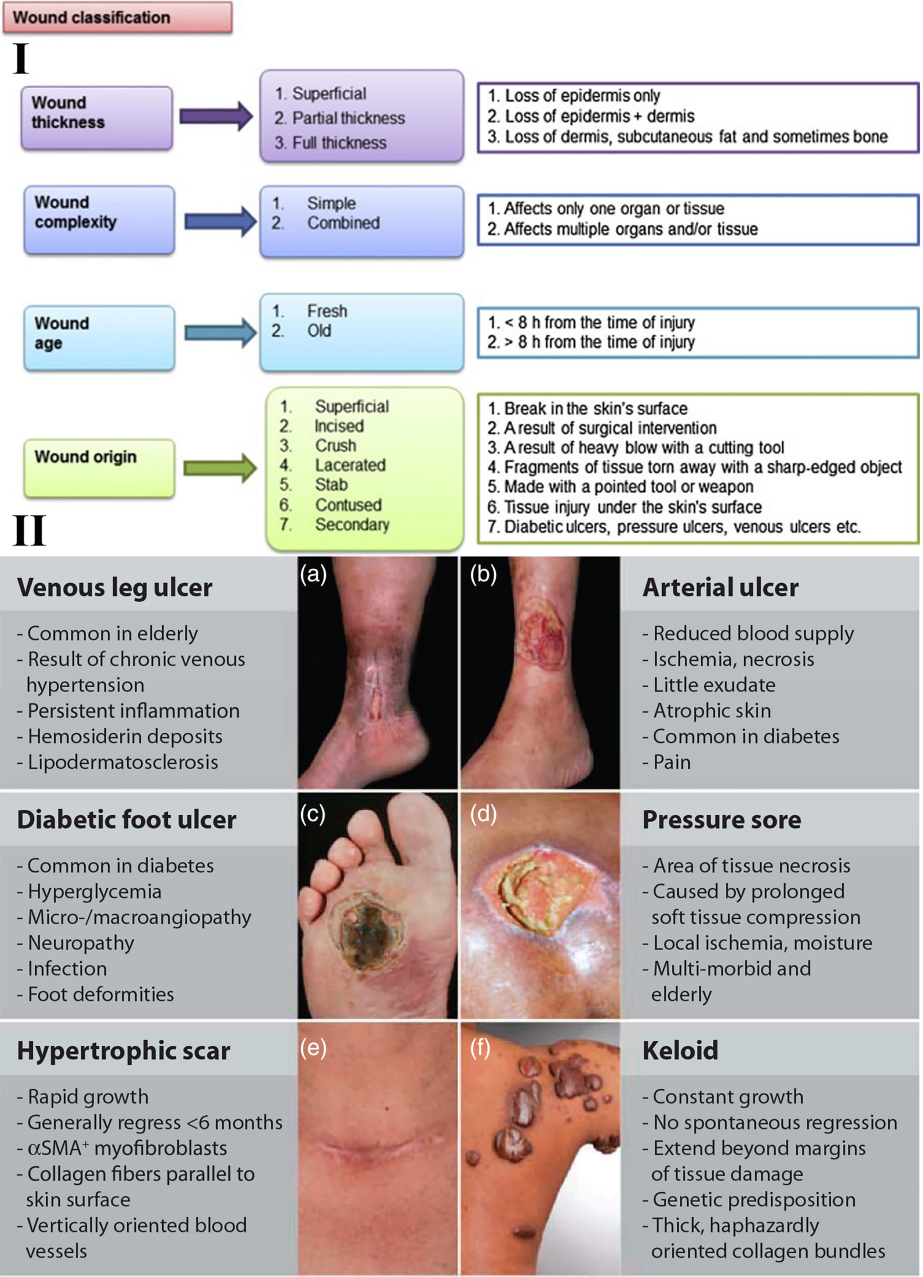
Wound classifications. I. Wound classification is based on thickness, complexity (simple or combined), age (fresh or old), and origin of occurrence. Reproduced with permission from Reference [86]. II. The most common wound healing pathologies are clinical features. A number of local and systematic factors leading to multiple wound‐healing pathologies may affect the repair process. (A) Medial aspect of the lower leg with venous leg ulcer (VLU). (B) Lateral aspect of the lower leg with an arterial ulcer. (C) Diabetic foot ulcer (DFU). (D) Pressure sore. (E) Hypertrophic scar after thyroid surgery. (F) Keloid. Reproduced with permission from Reference [87]

Skin is the largest organ in the body, because it covers the entire body surface area (about 2 m^2^), and protects it against disease and infection. Various factors such as accidents or diseases can result in skin damage or dysfunction, resulting in vulnerability to further trauma, germs, toxins, and infectious parasites.^84^, ^85^ Wounds can be categorized into acute or chronic based on the length of time they have been present, and their likelihood to be repaired by normal skin regeneration. It is possible for acute injuries causing either superficial or deep wounds to repair completely within 21 days, and little to no scar tissue formation is usually observed. In contrast, chronic wounds usually develop when acute wounds fail to heal even after 3 months. Diabetic wounds, which are common in individuals suffering from diabetes, are considered to be chronic. A more complete classification of various wounds is represented in Figure 10.^86^ The most important but challenging issue in wound treatment is to recover the tissue function with an acceptable aesthetic outcome in a short period of time. Large wounds especially in unhealthy condition such as diabetes, infectious disease and cancer can be very difficult to recover.

**FIGURE 11:**
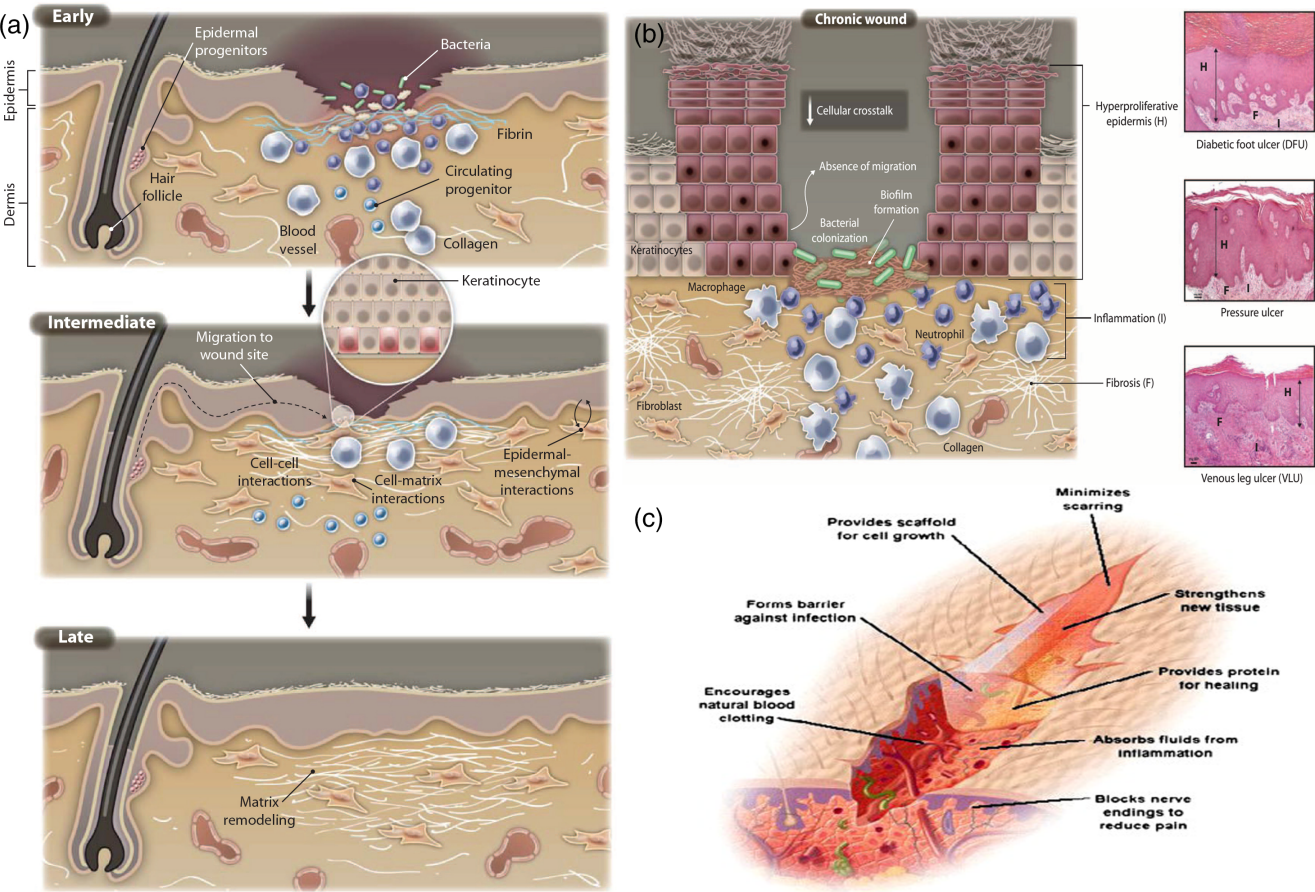
Molecular and cellular processes for the repair of normal skin. Molecular and cellular pathways for wound healing progression. The first step of wound healing comprises hemostasis and keratinocyte activation and inflammatory cells. The intermediate step includes keratinocytes, fibroblast proliferation, matrix deposition, along with angiogenesis. This spatiotemporal cycle is highly governed through several cell types that release various growth factors, cytokines, and chemokines to obtain wound closure and functional restoration. The chronic wound molecular pathology. Illustrations show the cellular and molecular mechanisms that are damaged in severe injuries. Although the increased inflammatory cells such as macrophages and neutrophils, they do not all work perfectly (validated using misshaped cells). Most fibroblasts mature into senescent ones. For chronic wounds, angiogenesis is decreased, the recruitment and activation of stem cells and the remodeling of ECM relative to the healing of injuries with the normal rate. Histology reflecting features of a diabetic foot ulcer (DFU), venous stasis ulcer (VLU), and bad sore. Such chronic wounds, though different etiology, have specific cellular characteristics depicted in (A): H, hyperproliferative epidermis; F, fibrosis, increased cellular inflammation.^87^ (C) Schematic representation of the required properties of a wound dressing material. Reproduced with permission from Reference [88]

Inadequate wound healing or excessive scar formation can affect many people after surgery, major trauma, or disease. There is still an incomplete understanding of the cellular and molecular mechanisms underpinning tissue repair, the failure of wounds to heal, and current therapies are limited. Even in the present day, the poor rate of wound healing after surgery, trauma, chronic injuries, or severe diseases, impacts many people all around the world. Insufficient attention paid to the key factors involved in tissue regeneration, including, inflammatory effects, matrix deposition, adequate angiogenesis, and cell recruitment could explain some of the difficulties faced with adequate wound healing. Additional clinical conditions, such as advanced age, cardiovascular disease, or diabetes are often associated with poor wound healing, because of the disturbances they cause to normal cellular functions. A deep insight into the fundamental biology of tissue restoration and regeneration could be useful to identify new therapeutic approaches to enhance the body's natural wound healing ability. Figure 10 illustrates the intricacy of wound healing involving several kinds of cells. Normal healing of acute wounds takes place in several overlapping phases, including the initial inflammatory response, deposition of the matrix, cell proliferation, cell migration, and tissue remodeling (Figure 10). Nonhealing chronic wounds are the consequence of an interruption in or deregulation of one or more wound healing stages (Figure 11B). The first thing to happen is the rapid activation of the coagulation cascade to produce a fibrin clot, which the provides the essential matrix architecture for recruiting inflammatory and other cell types into the wound. Platelets immobilized within the fibrin clot secrete localized growth factors and chemokines into the wound vicinity in order to attract other cells. The importance of platelets for effective wound healing has been demonstrated by the therapeutic application of platelet‐rich plasma rich in preclinical studies of wound healing,^89^ and the incidence of delayed healing in disorders related to platelets.^90^ In addition to hemostasis, the early inflammatory responses (both local and systemic) activate wound healing responses (Figure 11A). Inflammation is more pronounce in deep and more severe injuries (Figure 11B), It is thought that the chronic inflammatory state of such injuries can fail to resolve, thus, producing chronic wounds.^91^ More specifically, recent studies using molecular analysis of severely wounded tissue and wound fluid have shown a continuous competitive environment involving both pro‐inflammatory and anti‐inflammatory signals.^91^, ^92^ An increased number of neutrophils and macrophages are major components of the pro‐inflammatory cellular infiltrate that contributes to the delayed healing of chronic ulcers.^93^ These cells secrete primary pro‐inflammatory cytokines, including IL‐1β and tumor necrosis factor‐α (TNFα), which have been shown to prolong the inflammatory process and thereby delay healing.^92^, ^94^ Increased IL‐1β and TNFα in chronic wounds have been shown to lead to an increase in metalloproteinase enzymes that cause excessive local degradation of the ECM, and therefore prevent the migration of other cells designed to heal the wound.^95^ Recent studies have involved the inflammasome, a multiprotein complex produced by the innate immune system that continuously activates and secretes IL‐1β and IL‐18 inside chronic wounds.^96^, ^97^ Furthermore, the ongoing presence of a relatively high load of bacterial cells within chronic wounds (colonization) also continuously attracts pro‐inflammatory cells resulting in delayed wound healing (Figure 11).^87^ A more complete insight into the mechanisms regulating the inflammatory response is required before better approaches to the healing of chronic wounds can be devised.^87^

The ideal wound dressing should provide a suitable milieu for accelerating wound healing, minimizing the scar, increasing cellular activity (i.e., epithelial spreading, angiogenesis, and connective tissue synthesis). Wound dressings should also absorb wound exudate, maintain the proper moisture content and temperature within the wound (which also facilitates blood circulation and epithelial migration), exchange nutrients and oxygen, reduce pain, protect the wound against infection, and be nonadherent to the wound (i.e., easy to remove the wound dressing after healing). Moreover, dressings should encourage leucocyte migration, facilitate the activity of enzymes that can carry out debridement, and must also be sterile, nontoxic, and nonallergic.^84^ Table 2 present the guidance for wound management and some synthetic commercial wound dressings available on the market are summarized in Table 3.

**TABLE 2:** Wound management dressing guide

Type of tissue in the wound	Goal	Dressing performance	Wound area preparation	Primary dressing	Secondary dressing	Ref.	
Necrotic, black, dry	Cutoff devitalized tissue, do not try debridement if vascular insufficiency suspected, remain dry and refer for vascular assessment	Hydration of wound bed, stimulate autolytic debridement	Surgical/mechanicaldebridement	Hydrogel	Film dressing	98, 99
Sloughy, yellowish, brown, or black. Dry to low exudate	Eliminate slough, clean wound bed for granulation tissue	Rehydrate wound bed, adjust moisture balance, Stimulate autolytic debridement	Surgical/mechanical debridement	Hydrogel	Film dressing, low adherent (silicone) dressing	100, 101
Sloughy, yellow, brown, black or gray Modest to high exudate	Eliminate slough, rinse wound bed for granulation tissue, controlling exudate	Absorb exudates, Protect peri‐wound skin to avoid maceration, Stimulate autolytic debridement	Surgical/mechanical debridement	Moisture absorbent dressing, For deep wounds, utilize cavity strips	film dressing	102, 103
Granulating, clean, red Dry to low exudate	Boost granulation offer healthy wound bed for epithelialization	Maintain moisture balance Protect new tissue growth	Wound cleansing	Hydrogel, Low adherent (silicone) dressing, for deep wounds use cavity strips, rope or ribbon versions	Pad, avoid bandagesthat may cause occlusion and maceration. Tapes should be used with caution due to allergy potential and secondary complications	104, 105, 106
Granulating, clean, red moderate to high exudate	Controlling the exudate, offer healthy wound bed for epithelialization	Maintain moisture balance, Protect new tissue growth	Wound cleansing consider barrier products	Absorbent dressing, low adherent (silicone) dressingFor deep wounds, utilize cavity strips		
Epithelializing, red, pink, no to low exudate	Boost epithelialization and wound maturation	Protect new tissue growth		Hydrocolloid (thin), film dressing, low adherent (silicone) dressing		
Infected low to high exudate	Decrease bacterial load controlling the exudate controlling the odor	Antimicrobial performance moist wound healing odor absorption	Wound cleansing (consider antiseptic wound cleansing solution) consider barrier products	Antimicrobial dressing		

**TABLE 3:** Synthetic commercially available wound dressings

Wound dressing	Material	Function and Application	‐Advantage	Disadvantage	Company/brand name	Ref.
Hydrocolloid	Bilayer structure inner layer: gelatin, pectin, carboxymethyl cellulose, elastomers outer layer: waterproof layer	Maintains Moisture Low or medium exudative wound: pressure, diabetic wounds	Could be removed without pain, maintains the wound moisture adheres to the dry and humid part	Not suitable for highexudation wounds. Useless for infection wound and necrotictissues	3 M,Tegaderm, comfeel	107, 108
Hydrogel	Methacrylates, PVP, gelatin, pectin, chitosan	Donate moisture chronic dry wound (low humid), wound with necrotic tissue, mediumor deep wounds: low burning, pressure, and abrasion wounds	Provides a humid environment cools the wound and lowers pain. Useful for necrotic and infectious wounds	Dehydration, low absorbance	Aquaclear, purilon gel, hypergel sterigel	109
Alginate	Sodium/calcium alginate salts	Absorb exudate High and medium exudative wound,deep wound: diabetic, pressure, abrasion, venues, burning type II	Provides a humidenvironment, low allergic feature, useful for necrotic and infectious wounds	Dehydration, periodic control for deep wound	Algisite, algoderm, sorbsan	110
Foam	Silicone or PU foam	Absorb exudate high and medium exudative wound, diabetic, pressure, abrasion, venous, burning type II	Insulates heat and warms the wound, make pressure for venous ulcer	Allergic for delicate skin, useless for the dry and infectious wound	Allevyn, lyfoam, tegafoam	111
Semipermeable film	Transparent and semi‐permeable PU	Maintains Moisture Used as a second layerfor hydrogels and foams, nonexudative wound: surgical wound	Waterproof layer, permeable to oxygen, vapor. Barrier against infections	Useless for bloody/high exudative, necrotic, infectious wound	Tegaderm, hydrofim, polyskin	112
Antimicrobial dressings	Ionic silver, activated charcoal, Molecular iodine PHMB	Antibacterial	It can be used as different type of wound dressing such as Hydrofiber, Hydrocolloid and Gel or paste	It is not general dressing and based on wound the proper one should be selected	Smith & Nephew, Convatec, Coloplast Corp.,	113

The intrinsic properties of polylysine can play a role in the design of wound dressings. Polylysine has good biocompatibility together with pronounced antibacterial activity making it useful in wound dressings. Wang et al. synthesized a hydrogel derived from a combination of polyamino acids including ε‐polylysine and polyglutamic acid, and tested it as a wound dressing. This hydrogel had good biocompatibility, and showed antibacterial effects against both Gram‐positive and ‐negative bacteria, along with low inflammatory responses.^114^ Zou et al. synthesized a hydrogel wound dressing based on ε‐polylysine and carboxymethyl chitosan, which exhibited inherent antibacterial activity along with tunable properties, such as the swelling ratio (varying from 800% to 2000%) and the Young's modulus (varying from 10 to 100 kPa).^80^ Zhou et al. synthesized a photopolymerized hydrogel based on polylysine and methacrylamide, which exhibited good antibacterial and antifungal activity. Moreover, this hydrogel was also found to be non‐hemolytic and biocompatible.^115^

The closure of surgical incisions and tissue flap reattachment using suturing or staples, can be limited by incomplete sealing, scar formation, and leaving the possibility of bacterial infection. Alternative methods of tissue closure, for example, fibrin glue or cyanoacrylate glue have been developed to seal and reattach tissue flaps. These adhesives benefit from several advantages over conventional methods, but there are also shortcomings, such as, inadequate adhesive properties, possibility of infection, and concerns about possible toxicity. A new medical adhesive was designed based on oxidized dextran combined with ε‐PL, which possessed low cytotoxicity and enhanced adhesive strength for sealing tissue. The adhesive is activated to become adherent by absorbing water from bodily fluids, and can seal surgical incisions completely. Experimental results revealed that the sealing performance of this adhesive was superior to fibrin glue.^116^

Wang et al. synthesized an in‐situ gelling bioadhesive hydrogel based on ε‐polylysine for potentially infected wounds. Hydroxyphenylpropionic acid (HPA) was grafted onto an ε‐PL backbone and then cross‐linked using horseradish peroxidase (HRP) and hydrogen peroxide (H_2_O_2_). It was reported that the adhesive strength of the bioadhesive hydrogel (10–35 kPa) was higher than that of fibrin glue. Moreover, this platform showed good biocompatibility and antibacterial activity against both Gram‐positive and ‐negative bacteria. In‐vivo studies showed that the platform possessed an anti‐infective properties.^117^ Self‐healing properties were introduced into the hydrogel by adding plasma amine oxidase to the ε‐PL, because of the formation of Schiff base reaction products.^118^

**FIGURE 12:**
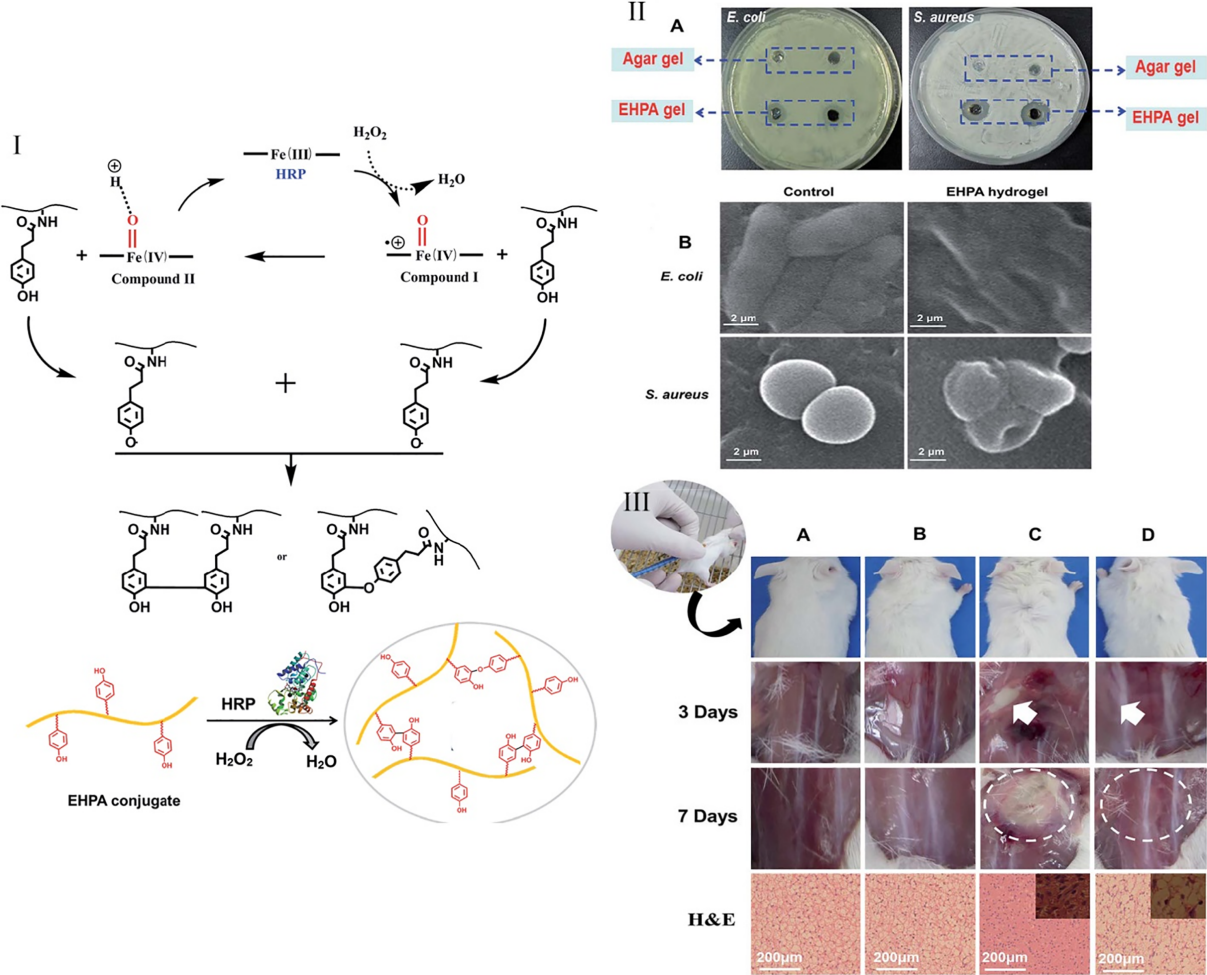
Antimicrobial effects of EHPA hydrogel. I. The proposed EHPA hydrogel‐forming mechanism through the oxidation reaction mediated by enzymes. II. Antibacterial activities against pathogenic Gram‐positive (*Staphylococcus aureus*) and ‐negative (*Escherichia coli*) bacteria by EHPA hydrogels (A) Antimicrobial ability of EHPA on agar through inhibition zones after 24 h. (B) SEM images of control sample of bacteria and after incubation in EHPA hydrogels. III. In vivo study. Images of the mice administered subcutaneously after 72 h and a week (*n* 1/4 3) with 10(8) CFU ml^−1^ *S. aureus* and histological analysis of inflammatory response to *S. aureus*, or *S. aureus* hydrogel, after a week (*n* 1/4 3). (A) Untreated (control), (B) treated with hydrogel, (C) treated with *S. aureus* and (D) hydrogel *S. aureus*‐treated groups. Hydrogel: 10 wt% EHPA crosslinked with HRP (0.05 mg ml^−1^) and H_2_O_2_ (0.06 wt%). Reproduced with permission from Reference [117]

As mentioned above, ε‐PL attaches to the bacterial surface destroying the cell membrane and causing cell lysis, resulting in cell death. SEM images were used to investigate the antimicrobial mechanism of ε‐PL, by visualizing morphological changes occurring during cell‐ ε‐PL interactions. As shown in Figure 12, rodlike (*E. coli*) and round (*S. aureus*) cellular morphology was preserved when the bacteria were cultured on a control hydrogel. By contrast, a disrupted and withered surface was observed for bacteria cultured on EHPA hydrogels.^117^ *S. aureus* is a well‐known species that contributes to many wound infections. This bacterial species was embedded in EHPA hydrogels, which were then subcutaneously implanted in a mouse model to assess its potential as an antimicrobial hydrogel in clinical application. Sterile saline, noncontaminated EHPA hydrogels, *S. aureus* bacteria alone, and bacteria loaded‐EHPA hydrogels were all injected into groups of mice to evaluate the effects of the hydrogel on infection prevention and treatment. Three days after injection with bacteria alone, the positive control mice were close to death. The animals were divided into two groups; one group was sacrificed and dissected to observe the infected tissue. As shown in Figure 12, in the mice injected with bacteria alone, an abscess was observed. On the other hand, in the mice injected with bacteria loaded‐EHPA hydrogels, no infection was observed. In the sterile saline‐injected mice, the tissue morphology was similar to control mice. No pathological signs were observed for sterile hydrogel‐injected mice, showing the biocompatibility of the hydrogel after 7 days. Hematoxylin and eosin (H&E) staining showed widespread bacterial infection that could be prevented by the EHPA hydrogel (see Figure 12). Furthermore, an inflammatory response was also observed in tissue infected with bacteria alone, with numerous inflammatory cells migrating to the injection site. However, injection of the bacteria‐loaded EHPA hydrogel, sterile EHPA hydrogel, or sterile saline resulted in no inflammation (Figure 12).^117^

## 6 POLYLYSINE FOR THERAPY OF SKIN DISEASE

The design and preparation of systems for the delivery of nucleic acids and therapeutic genes has gained attracted interest in recent years. Gene therapy strategies can be used for the treatment of many diseases, such as cancer, inherited disorders, viral infections, and wound management, especially in diabetic patients. Non‐viral gene delivery systems (nVGDS) can be used in gene therapy, where gene expression is promoted in fibroblasts to improve skin wound healing.^119^

Various virus‐based vectors (e.g., retroviruses, adenoviruses, and adeno‐associated viruses) and also nonviral vectors (e.g., lipoplexes, polyplexes, and plasmid DNA) have been tested to deliver genetic material to target cells.^120^ nVGDS have attracted much interest because they do not suffer from the safety issues related to viral vectors. nVGDS can enable the efficient transfer of genes to the intracellular compartment of cells. nVGDS based on polycations (e.g., polyethyleneimine [PEI]) which benefit from high transfection efficiency and low toxicity, are effective gene delivery platforms both in vitro and in vivo. However, ex vivo gene therapy (i.e., extraction of cells, genetic modification, and re‐transplantation of the modified cells) might benefit from the fact that the cell culture step gives better control of the whole process. Furthermore, exposure of the body to nVGDS is significantly reduced, resulting less risk of undesirable side effects. The transfer of foreign DNA into the cells, involves a multistep process essentially relying on endocytosis.^121^ An endocytosis inhibitor brefeldin reduced DNA delivery mediated by many cargo‐vehicles, such as, PEI, adenovirus, and Lipofectamine‐2000.^92^ All the non‐viral carriers are designed to package exogenous DNA (EDNA) or nucleic acids into a biocompatible complex which can easily be transported and adsorbed into the cells. After endocytosis into the cells, the EDNA will be contained inside endosomes and lysosomes, because these organelles are designed to break down foreign substances and microorganisms. The problem is then how to transfer these nucleic acids into the cytosol, and then into the nucleus without being degraded by hydrolytic enzymes.^122^ Nuclear pores can allow certain nucleic acid sequences to penetrate into the nucleus.^123^ However, the DNase II enzyme present in the extracellular space and in the cytoplasm is able to degrade DNA. The success of gene therapy using non‐viral vectors depends on their ability to protect the nucleic acids against degradation by nuclease enzymes, as well as to allow cell uptake.^124^

Abbasi et al. conjugated palmitic acid (PA) to PLL to form a vehicle to transfer a plasmid DNA coding for enhanced green fluorescent protein (pEGFP) into skin fibroblasts. PLL‐PA was found to give the highest number of EGFP positive cells. EGFP is a frequently used reporter gene to measure the transfer of plasmids (pEGFP) for skin tissue regeneration. In addition, the relative efficiencies of various lipid‐substituted poly‐l‐lysines were studied. According to their results, the resistance to dissociation after heparin treatment was related to the degree of lipid substitution in the polymer composite. Lipid‐substituted PLLs exhibited the best complex formation with pEGFP. These polymers also showed good protection of pEGFP from in vitro digestion by DNase I and DNase II. Upon delivery by lipid substituted polymers, the intracellular pEGFP expression was stable for up to 7 days. Additional investigation using flow cytometry showed that modification of PLLs with stearic and myristic acid allowed successful protein expression, and myristic acid showed the lowest toxicity. They found that compared to pristine lipid alone, lipid conjugated PLL led to more efficient gene delivery.^125^

Impaired angiogenesis is a significant clinical concern that might explain delayed wound healing, particularly in diabetes patients. One strategy to accelerate wound healing in diabetes patients, used an approach which could enhance angiogenesis. There were two peptides involved: (1) the TG peptide was covalently linked to the fibrin network for sustained release; (2) a polyR peptide was used to enhance the cellular uptake of these nanocondensates. To induce angiogenesis, both modified and unmodified polymers were condensed with plasmid‐DNA in vitro. HIF‐1α is an oxygen‐sensitive transcription factor that contains a domain for oxygen‐dependent degradation (ODD), and HIF‐1α is naturally degraded under normoxic conditions. The temporary‐induced expression of the angiogenesis‐linked genes Acta2, Pecam1, along with HIF‐1α and VEGF was observed after PLL‐g‐PEG polymer‐mediated DNA transfer of a HIF‐1α − ΔODD plasmid. In addition, the delivery of the modified *HIF‐1α* gene improved wound healing and increased the number of endothelial and smooth muscle cell precursors making more mature blood vessels.^126^

Erlach et al. synthesized poly(2‐methyl‐2‐oxazoline)‐grafted poly(l‐lysine) particles as a nonviral delivery vehicle for therapeutic DNA (TDNA) for improved wound healing. The polyplexes were soluble in serum and after heating to 70°C, and they preserved the condensed DNA from digestion by DNase‐I. The efficacy of DNA‐PMOXA‐g‐PLL DNA transfection was strongly dependent upon the number of PMOXA molecules grafted onto the polymer chains; a low grafting density between 7% and 14% and a medium N/P ratio (3.125–6.25) was found to be the best. As an alternative to PLL‐g‐PEG‐DNA polyplexes formed from PLL20‐g7PMOXA4 may be promising for the transfection of TDNA.^127^

## 7 TRANSLATIONAL SUCCESS AND CHALLENGES

Translational research tries to generate meaningful, applicable outcomes that advance human health and translate fundamental science findings more efficiently into practical usages. It is worth mentioning that healthcare is an important subject in translational research, particularly in using advanced materials. Different type of advanced biomaterials have been used as tissue replacements, regenerating scaffolds, drug carriers, and releasing vehicles known as a translational approach. Among these biomaterials, Polylysine can be engineered in different structures to be used in tissue engineering and as a drug/gene carrier. It is reported that polylysine/CAG‐peptide is used for the clinical treatment of cardiovascular diseases.^128^ Moreover, polylysine/chitosan is proposed as a proper substrate for clinical usage as an anti‐hemorrhaging hydrogel.^129^ However, it is reported that the polylysine show toxicity in some cases, and therefore it should be controlled for the desired application, which is known as a translation challenge.^130^ It can be deduced that the successful translational of polylysine is a subtle balance between simplicity and complexity. Hence, the polylysine‐based products should be designed based on final application to achieve the proper results.

## 8 CONCLUDING REMARKS AND FUTURE PERSPECTIVES

**FIGURE 13:**
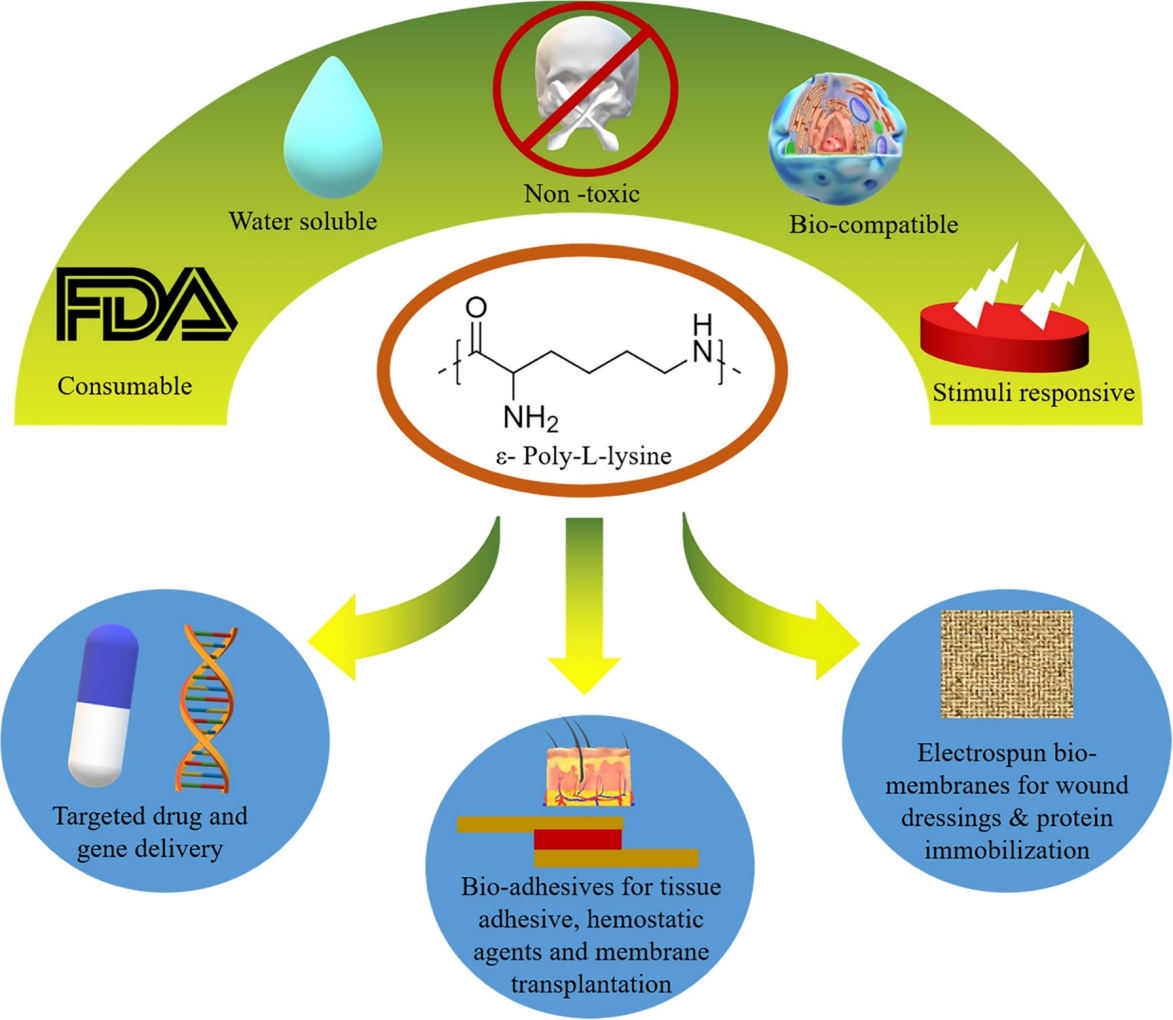
Future perspective and desired performance of the polylysine. Reprinted with permission from Reference [131]

Polylysine can be used as a biomaterial to improve wound healing and skin regeneration due to its beneficial features. Several promising results have been obtained based on the preclinical and clinical experiments made in the past years. Herein, we have summarized several instances of polylysine and its composites being used as antimicrobial materials to assist wound healing and act as a gene delivery vector to treat skin diseases. Polylysine could be applied in different formulations: scaffolds, gels, dressings/films, or nanoparticles because of its stimuli‐responsive performance, biocompatibility, and FDA approval (Figure 13).

Suppose everything goes well with polylysine usage in wound management; in that case, it is expected that the polylysine scaffold shows proper antibacterial effect along with proper cell adhesion and proliferation, which causes rapid wound healing. Moreover, as a gene delivery vehicle, polylysine is one of the most preferred carriers for gene delivery which can be tailored to play a suitable role in gene therapy. Despite some promising results with formulations based on polylysine obtained in skin regeneration and wound healing, several challenges remain. For example, preparation methods and characterization techniques should be optimized and standardized. Adjusting the suitable surface properties, design, and pore size required by cells to proliferate and principally to differentiate with the right phenotype is also another challenge. Drug delivery applications should be tailored to biological mechanisms involved in wound healing, and thus, considering the essential role of proper vascularization is another challenge in this realm. Moreover, gene transfer applications should be further investigated to better control the proton sponge effect for lysosomal escape.

## CONFLICT OF INTEREST

Michael R. Hamblin declares the following potential conflicts of interest. Scientific Advisory Boards: Transdermal Cap Inc, Cleveland, OH; Hologenix Inc. Santa Monica, CA; Vielight, Toronto, Canada; JOOVV Inc, Minneapolis‐St. Paul MN; USHIO Corp, Japan; Sanofi‐Aventis Deutschland GmbH, Frankfurt am Main, Germany.

## AUTHOR CONTRIBUTIONS

**Payam Zarrintaj:** Conceptualization (lead); investigation (equal); project administration (equal); writing – original draft (lead); writing – review and editing (equal). **Sadegh Ghorbani:** Data curation (equal); investigation (supporting); writing – original draft (supporting). **Mahmood Barani:** Data curation (supporting); writing – original draft (supporting); writing – review and editing (supporting). **Narendra Pal Singh Chauhan:** Writing – original draft (supporting); writing – review and editing (supporting). **Mohsen Khodadadi Yazdi:** Writing – original draft (equal). **Mohammad Reza Saeb:** Conceptualization (equal); writing – review and editing (supporting). **Joshua Ramsey:** Data curation (supporting); writing – review and editing (supporting). **Michael Hamblin**: Conceptualization (equal); data curation (supporting); supervision (equal); writing ‐ review & editing (supporting). **Masoud Mozafari**: Conceptualization (equal); data curation (equal); supervision (equal); writing ‐ review & editing (equal). **Ebrahim Mostafavi:** Conceptualization (equal); funding acquisition (lead); supervision (equal); writing – review and editing (equal).

### PEER REVIEW

The peer review history for this article is available at https://publons.com/publon/10.1002/btm2.10261.

## Data Availability

Data sharing not applicable to this article as no datasets were generated or analysed during the current study.
